# Key Competencies in Emergency Medicine Training in Belgium: A New Criteria-Based Evaluation Strategy

**DOI:** 10.7759/cureus.70772

**Published:** 2024-10-03

**Authors:** Isabelle Kong Kam Wa, Yves Maule, Fabien Guerisse

**Affiliations:** 1 Emergency Department, Centre Hospitalier Universitaire Brugmann, Brussels, BEL; 2 Public Health Department, University of Liège, Liège, BEL; 3 Emergency Department, Centre Hospitalier Universitaire Charleroi-Chimay, Charleroi, BEL

**Keywords:** competencies, criteria-based assessment grid, delphi method, dreyfus model, emergency medicine, postgraduate curriculum, skill acquisition

## Abstract

Background

In French-speaking Belgium, Emergency Medicine (EM) has been a recognized professional qualification for the last two decades. Currently, there is no consensus on the core competencies required for EM postgraduates. During the six-year training period, the acquisition of technical skills (scientific knowledge and technical procedures) is emphasized, but little account is taken regarding the development of non-technical skills such as communication, collaboration, leadership, and professionalism. Furthermore, currently, there is no criteria-based assessment grid to evaluate the achievement of skills at the end of each three-month clinical rotation. The subjectivity inherent in the current evaluation process increases the inequity and variability from one clinical rotation to another. This study aimed to develop a practical tool: a criteria-based assessment grid to identify the core competencies required of EM postgraduates, help learners identify skills that have been acquired and those not yet attained, and establish an evaluation process that is equitable, objective, and uniform.

Methodology

Using a group facilitation technique, we developed a consensus among a panel of expert emergency department clinical rotation supervisors from three French-speaking universities. During the first phase of the study, the experts validated a list of core competencies adapted from different existing competency frameworks. The validated core competencies constituted the evaluation criteria for the assessment grid. During the second phase of the study, the experts determined the level of skill expertise expected for each of these competencies, depending on the training level of the EM postgraduate. In our assessment grid, five levels of skill expertise and three levels of EM postgraduate training were defined.

Results

A total of 18 Emergency department clinical rotation supervisors from the three French-speaking universities participated during the first round of the process, 12 during the second round, and 11 during the third round. Of the 81 initial competencies proposed, 78 reached consensus. For each of these competencies, the level of skill expertise was determined for the three levels of training.

Conclusions

A criteria-based assessment grid was developed by consensus. The grid identifies the core competencies required of EM postgraduates, integrating technical and non-technical skills. Used at the end of each clinical rotation, it determines which skills have been acquired and where gaps exist, allowing for improvements in EM training. The assessment grid promotes an evaluation that is criteria-based, objective, and uniform from one clinical rotation to another.

## Introduction

In French-speaking Belgium, Emergency Medicine (EM) has been recognized as a professional medical qualification only since 2005 [1]. The residency program, known also as a postgraduate in EM, follows a standard time-based model, where postgraduates devote 72 months to clinical training before completing their specialization. Postgraduates rotate every three to six months from one clinical department to another, and during each rotation period, are under the supervision of a local clinical rotation supervisor (who is generally the head of the department). Currently, there is no consensus on the core competencies required for EM doctors to achieve during their six-year postgraduate training. Qualitative knowledge and technical skills needed to perform adequately in emergency departments are acquired, but little is taught about non-technical skills such as communication, collaboration, and leadership. Furthermore, at the end of the six-year curriculum, students may graduate without having mastered all the required skills. Each EM postgraduate is assessed by the local clinical rotation supervisor every quarter. Evaluation is based upon an overall appreciation, from excellent to poor. We consider that the subjectivity inherent in the process is a core problem that increases the inequity and the variability from one clinical rotation to another. As we write this paper, in Belgium, there is no criteria-based assessment grid to evaluate the achievement of skills at the end of each clinical rotation.

Therefore, we identified a great need to determine the competencies required for Belgian EM postgraduates. It is important to emphasize the acquisition of technical and non-technical skills during the six-year training period. The development of these “soft skills” is crucial in today’s highly demanding society, enabling better communication, reducing patient aggressiveness, and increasing patient satisfaction [2]. It is also urgent to establish an equitable assessment from one clinical rotation to another by reducing the subjectivity inherent in the process [3]. Students might benefit from better quality feedback, leading to greater satisfaction in the postgraduate curriculum; objective assessment ensures greater fairness in the evaluation process. In addition, iterative supervision might identify at an early stage students struggling with their skills and in need of greater support [4].

The objective of this study was to create a referential assessment tool that defines the core competencies required of all EM doctors, identifies the postgraduate’s progression during the six-year training period, and promotes an evaluation that is criteria-based, objective, and uniform from one clinical rotation to another.

## Materials and methods

Constructing a criteria-based assessment grid required determine beforehand the following three steps [5]: defining the assessment criteria (the core competencies required of EM postgraduates); defining the training levels of EM postgraduates (to determine learner progression from one level to the next); and defining the levels of skill expertise (for each core competence).

First step: definition of the assessment criteria

To define the assessment criteria (the core competencies required of EM postgraduates), we based our study on two existing competency frameworks, i.e., the CanMEDS Physician Competency Framework, developed by the Royal College of Physicians and Surgeons of Canada [6,7], and the core competencies framework developed by the French Society of Emergency Medicine (Référentiel métier-compétences pour la spécialité de médecine d’urgence) [8]. CanMEDS is a guide that describes the competencies that all doctors must acquire and integrate to provide high-quality care and meet patients’ needs [6]. Since the beginning of the 21st century, it has formed a part of a worldwide movement that promotes the implementation of a new model in medical education, competency-based medical education (CBME) [9]. CanMEDS describes seven roles, with the central role being Medical Expert, and six other roles, namely, Communicator, Collaborator, Leader, Health Advocate, Scholar, and Professional. Key competencies are defined for each of these roles. In 2018, core competencies were defined for Canadian EM specialists and training programs shifted to a competency-based education model [10]. The French core competencies framework for EM also describes the skills and behavioral aptitudes required of an EM doctor [8]. This competency framework was developed for the specialization in EM, officially recognized as a specialty in France in 2015 [11]. It describes the missions of the EM doctor and the laws regulating the practice. Required skills are listed for different clinical syndromes, including theoretical knowledge and technical and behavioral skills. By hybridizing competencies from these two frameworks and adjusting them to the Belgian healthcare system, an initial list of 81 core competencies was first established.

Second step: definition of the emergency medicine postgraduate training levels

Skill acquisition develops in stages and continues throughout the professional career. A model of skill acquisition was developed in the 1980s by Hubert Dreyfus and Stuart Dreyfus (University of California, Berkeley) [12]. The model describes the following five stages of learning development: Novice, Advanced Beginner, Competent, Proficient, and Expert. It has been used and adapted by medical educators to establish frameworks of skill development in practical clinical medicine [13]. This concept does not exist in Belgian EM. In Belgium, postgraduates are distinguished according to their year of training. However, it is difficult to make a precise distinction between the acquisition of skills by a first-year and a second-year postgraduate. During the evaluation process, expectations are not the same when assessing a student at the beginning of their six-year curriculum than when they are at the end. For this study, an adapted Dreyfus and Dreyfus model of skill acquisition was developed, which we named “Levels of Training.” We decided to use the levels “Beginner,” “Competent,” and “Proficient.” “Novice” and “Expert” represent levels of skill acquisition that do not correspond to the postgraduate curriculum and were therefore inadequate for the study. The six years of postgraduate training were divided into the following three groups: first- and second-year students were considered “Beginners.” Third-, fourth-, and fifth-year students were considered to be “Competent.” Sixth (and last) year students were considered to be “Proficient.” The definitions established for each level of training are presented in Table 1.

**Table 1:** Levels of training. Definitions established of the three levels of EM postgraduate training adapted from the Dreyfus and Dreyfus model of skill acquisition [12,13]. EM: emergency medicine

Levels of training	Definitions
Beginner	A Beginner is at the start of clinical practice. A practical knowledge of the key elements of the discipline has been acquired, but holistic vision is still limited. To solve problems, actions have to be converted into a series of steps. Each aspect treated separately is considered with equal importance. The Beginner is capable of carrying out simple tasks, but requires supervision for synthesis work and needs to develop the ability to discern priorities. The complexity of the situation is perceived but can be resolved only partially. In Belgian EM, the Beginner is at the start of the postgraduate curriculum and is equivalent to a first and second year of specialization
Competent	A Competent has experienced a few years of clinical practice. A good knowledge of the discipline has been acquired; sufficient clinical judgment has been developed to resolve common clinical situations. The Competent is autonomous in carrying out most tasks and starts to perceive the globality of the situation; actions are conceived over the long term. Faced with complexity, an analytical approach is still used; problems have to be separated into a series of steps to be able to solve them. In Belgian EM, the Competent is in the middle of the postgraduate curriculum and is equivalent to a third, fourth, and fifth year of specialization
Proficient	Proficiency is the stage of autonomy. A Proficient perceives clinical situations holistically: the globality of the situation is discerned and how each aspect fits into the whole. Knowledge of the discipline is profound and problem-solving seems intuitive. The Proficient is autonomous, fully responsible, and delivers a high standard of care on a daily basis. Complex and evolving situations can be easily dealt with. In Belgian EM, the Proficient is at the end of the postgraduate curriculum and is equivalent to the sixth (and final) year of specialization

Third step: definition of the skill expertise levels

We defined the level of skill expertise for each core competence. For a given competence, an EM postgraduate at the beginning of the curriculum will not master the competence in the same way as one in the last year of specialization. In our study, we defined the following five levels of skill expertise: “Inactive,” “Observer,” “Apprentice,” “Functional,” and “Experienced.” These five levels, adapted from a pregraduate student assessment grid developed by the Faculty of Medicine of the Université Libre de Bruxelles (Grille d’évaluation des apprentissages développés en stage. Nouwynck, S. Université Libre de Bruxelles), were named “Levels of Skill Expertise.” The definitions established for these five levels are presented in Table 2.

**Table 2:** Levels of skill expertise. Definitions established of the five levels of skill expertise, adapted from a pregraduate student assessment grid developed by the Faculty of Medicine of the Université Libre de Bruxelles (Grille d’évaluation des apprentissages développés en stage. Nouwynck,S. Université Libre de Bruxelles). EM: emergency medicine

Levels of skill expertise	Definition
Inactive	The EM postgraduate is not proactive in training
Observer	The EM postgraduate has only theoretical knowledge of the activity and is not yet able to carry out the activity
Apprentice	The EM postgraduate is able to carry out the activity under supervision and requires assistance with explanations
Functional	The EM postgraduate is capable of carrying out the activity appropriately, according to the standard of care expectations
Experienced	The EM postgraduate is capable of carrying out the activity appropriately, according to the standard of care expectations, and can adapt to the specific aspects of each situation. The Experienced EM postgraduate is capable of explaining (and supervising) the activity to an Observer or an Apprentice

The modified Delphi process

**Figure 1:**
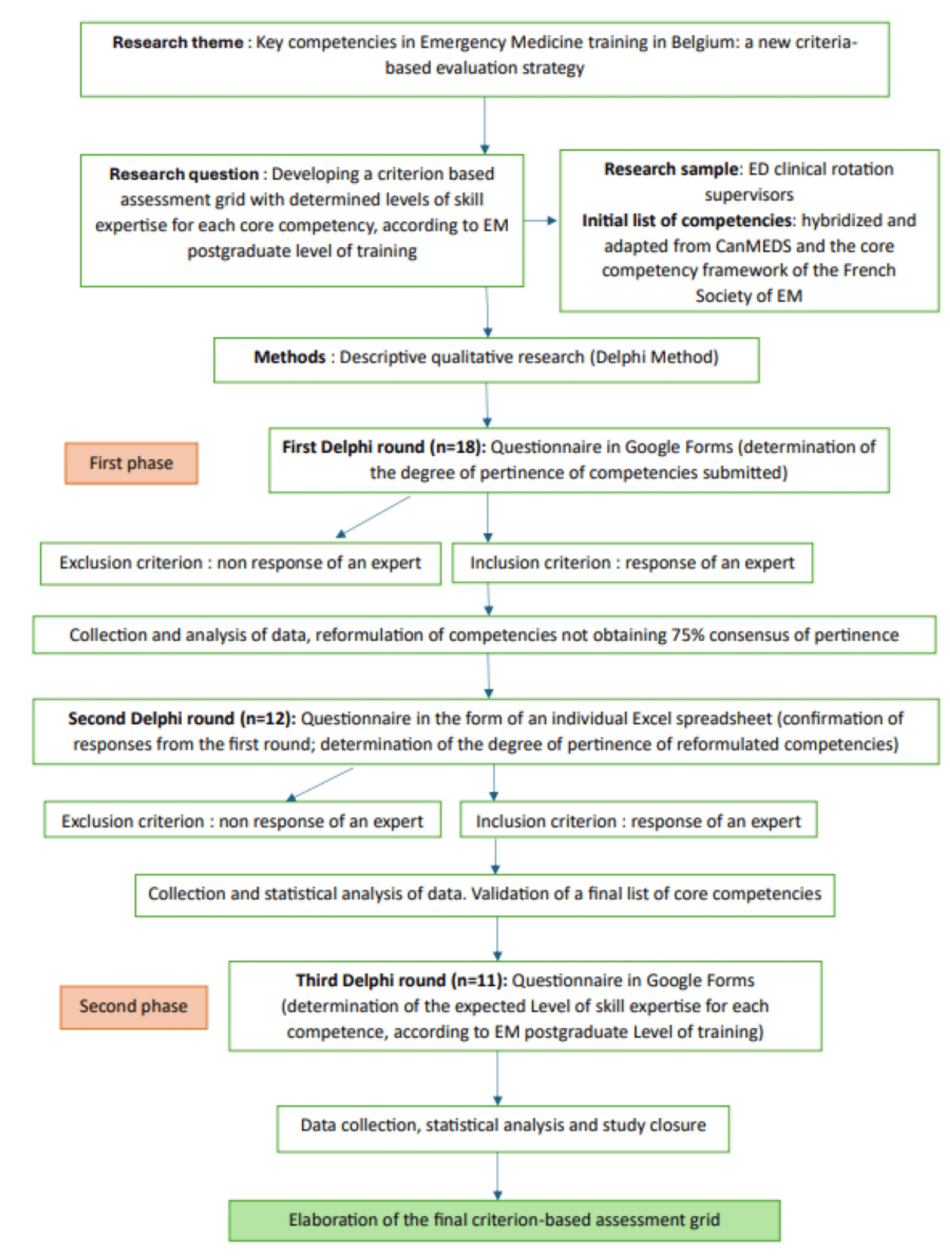
Study flowchart.

To elaborate our criteria-based assessment grid, a modified Delphi process was used [14-20]. We obtained consensus on the opinions of a panel of experts (emergency department clinical rotation supervisors). The plan for this study began in September 2023. The research sample consisted of emergency department clinical rotation supervisors from three French-speaking universities (Université Libre de Bruxelles, Université Catholique de Louvain, and Université de Liège), who are academic “Experts” responsible for the evaluation of EM postgraduates. The study was divided into two phases. The aim of the first phase (the first and second Delphi rounds) was to validate a list of core competencies for the assessment grid. During the second phase (the third Delphi round), for each of the validated competencies, the level of skill expertise was determined according to the training level of the postgraduate. The flowchart of the study is shown in Figure 1.

First Delphi Round

The first questionnaire, created in Google Forms, comprised the initial list of 81 core competencies hybridized and adapted from the CanMEDS competency framework and the core competencies framework of the French Society of Emergency Medicine. In January 2024, an invitation was sent by email to 30 emergency department clinical rotation supervisors of the three French-speaking universities. Participation was voluntary and the process was conducted in an anonymous manner (the identities of participants were not known between experts). This first questionnaire was divided into seven sections (for the seven CanMEDS roles), and within each section, a list of competencies was proposed. The experts were asked to rate, on a five-point Likert scale (from “Not at all pertinent” to “Very pertinent”), whether the competence was considered pertinent for inclusion in the final assessment grid. A free text zone was provided for eventual comments. The response deadline was two weeks with a reminder email sent after one week. The inclusion criterion consisted of a response from an expert. The exclusion criterion was non-participation. Competencies that obtained a consensus of 75% of pertinence (those considered “Pertinent” and “Very pertinent”) were retained. Competencies that did not achieve 75% consensus of pertinence were withdrawn and reformulated. The experts’ comments were taken into account.

Second Delphi Round

The questionnaire for this second Delphi round, in the form of an Excel spreadsheet, was sent individually to experts who responded to the first round. In February 2024, each expert received a personal Excel spreadsheet (thus guaranteeing anonymity), containing the group results, their individual responses from the first round, and the reformulated competencies. The participants were asked to confirm or modify their opinions in light of the group results and to indicate the degree of pertinence of each of the reformulated items. A two-week deadline was accorded, with a reminder email sent after one week. The inclusion criterion was the response of an expert, and the exclusion criterion was non-participation. Statistical analysis of the data using the RAND/UCLA Appropriateness Method (RAM) [21] was performed on the results of this second Delphi round: for each competence, the experts’ responses were ranked as follows: Very pertinent = 5, Pertinent = 4, Neutral = 3, Not very pertinent = 2, and Not at all pertinent = 1. The median, 30th (C30), and 70th (C70) percentiles of the distribution were calculated, as well as the interpercentile range (IPR) and the interpercentile range adjusted for symmetry (IPRAS). Pertinence and expert consensus were considered according to Table 3 [21].

**Table 3:** Interpretation of the results of expert responses (second Delphi round). Interpretation of the results of expert responses using the RAND/UCLA Appropriateness Method [21]. IPR: interpercentile range; IPRAS: interpercentile range adjusted for symmetry

Level of pertinence (median of the expert group)	Agreement of experts (IPR < IPRAS)	Disagreement of experts (IPR > IPRAS)
4-5 = Pertinent	Agreement of pertinence	Disagreement of pertinence
3 = Uncertain pertinence	Agreement of uncertain pertinence	Disagreement of uncertain pertinence
1-2 = Not pertinent	Agreement of non-pertinence	Disagreement of non-pertinence

A competence was validated if the median was 4-5 (pertinent) and the value of IPR < IPRAS (expert agreement on pertinence). A competence, however, was rejected in the following three situations: when the median was 3 (uncertain pertinence) and IPR < IPRAS (agreement of uncertain pertinence); when the median was 1-2 (not pertinent) and IPR < IPRAS (agreement of non-pertinence); when IPR > IPRAS (disagreement between experts). At the end of this second round, a list of core competencies was validated.

Third Delphi Round

The aim of this second phase of the study (and third Delphi round) was to determine the expected level of skill expertise for each of the validated competencies, according to the level of training of the EM postgraduate. Three levels of training were defined, adapted from the Dreyfus and Dreyfus model of skill acquisition: Beginner-Competent-Proficient. Five levels of skill expertise were defined, adapted from the pre-graduate assessment grid developed by the Faculty of Medicine of the Université Libre de Bruxelles: Inactive-Observer-Apprentice-Functional-Experienced. In March 2024, a new questionnaire in Google Forms was sent to the experts who had participated in the second round. The questionnaire listed the core competencies that had been validated at the end of the second round. For each competence, the experts were asked to determine the level of skill expertise expected of a Beginner, a Competent, and a Proficient. A free text zone was provided for eventual comments. The deadline for response was two weeks, with a reminder e-mail sent after one week. The inclusion criterion was the response of an expert, and the exclusion criterion was non-participation. Statistical analysis was performed on the data from this third round. The levels of skill expertise were ranked as follows: Inactive = 1, Observer = 2, Apprentice = 3, Functional = 4, Experienced = 5. The median value, first quartile (Q1), and third quartile (Q3) of each distribution were defined and the interquartile range (IQR) was calculated.

## Results

A total of 18 ED clinical rotation supervisors (out of 30) responded positively in the first Delphi round. During the second round, 12 experts (out of the 18) participated, and during the third round, 11 experts (out of the 12) participated. Three French-speaking universities were represented in each round.

Concerning the first round, 81 initial core competencies (from the seven CanMEDS roles) were submitted to the expert panel (n = 18). Of these 81 competencies, 74 reached consensus (75% of experts considered the competence to be “Pertinent” or “Very pertinent”). Seven competencies did not reach a consensus and had to be reformulated.

The 74 competencies that achieved consensus in the first round were confirmed in the second Delphi round. Their level of pertinence was “Pertinent” (median = 4-5), and the experts’ agreement was pertinent (IPR < IPRAS). Of the seven reformulated competencies, four reached consensus (median = 4-5 and IPR < IPRAS). Three reformulated competencies were of uncertain pertinence (median = 3) and had an expert agreement of uncertain pertinence (IPR < IPRAS). These three competencies, not reaching consensus, were therefore excluded. In total, 78 core competencies (from the seven CanMEDS roles) were validated by the experts (n = 12) at the end of the second Delphi round (Table 4).

**Table 4:** The 78 core competencies validated after the second Delphi round.

Role	Competencies
Medical Expert	1. Provide high-quality care to patients admitted to the emergency department
	2. Demonstrate appropriate knowledge relevant to Emergency Medicine concerning the anatomy, physiology, and pathophysiology of the various systems (cardiovascular, pulmonary, gastrointestinal, genitourinary, gynecologic, endocrine, neurological, musculoskeletal, hematologic, and immunologic systems)
	3. Demonstrate appropriate knowledge concerning the epidemiology of common diseases encountered in the emergency department
	4. Demonstrate appropriate knowledge relevant to Emergency Medicine concerning community and nosocomial infections, antibiotic stewardship, and antimicrobial prophylaxis
	5. Demonstrate appropriate knowledge concerning the management of immunocompromised and transplanted patients, who are at a high risk of complications
	6. Demonstrate appropriate knowledge concerning contagious diseases that are compulsory to declare and the reporting procedures specific to your region
	7. Demonstrate appropriate knowledge concerning toxicology relevant to Emergency Medicine
	8. Demonstrate appropriate knowledge concerning the pharmacology of medications used in emergency departments (analgesics, sedatives, antimicrobials, cardiovascular medications, thrombolytics, endocrine, respiratory and neuropsychiatric medications, recreational drugs)
	9. Demonstrate appropriate knowledge concerning the general management of polytraumatized patients and the mechanisms of injury
	10. Demonstrate appropriate knowledge concerning the management of critically ill patients, life-threatening situations, cardiopulmonary resuscitation, and pathologies requiring intensive care
	11. Demonstrate appropriate knowledge concerning the principles of pre-hospital medicine, management of environmental emergencies, and mass casualty and disaster medicine
	12. Demonstrate appropriate knowledge of the medico-legal concepts concerning the treatment of psychiatric patients, the protection of children, and other victims of abuse
	13. Demonstrate appropriate knowledge of the following concepts: limitation of the therapeutic effort, no escalation of treatment, and end-of-life care
	14. Be able to manage emergency department flow under normal circumstances, while carrying out other professional duties
	15. Realize triage care for multiple patients in crisis situations
	16. Be able to identify and treat priority patients with life-threatening conditions
	17. Elicit the patient’s clinical information in a concise, structured way: be able to prioritize the problems to be treated, research the patient’s psycho-social situation, and use other sources to complete clinical information
	18. Carry out accurate and complete physical and mental assessments, particularly concerning patients presenting non-specific clinical symptoms or syndromes
	19. Be able to identify and treat serious pathologies that are less commonly encountered
	20. Select appropriate investigation methods based on their diagnostic utility, paying attention to patient safety, available resources, and the cost-benefit ratio
	21. Interpret the results of investigation methods used (laboratory analysis, radiologic imaging, and electrocardiogram)
	22. Recognize that diagnostic uncertainty exists and be able to use relevant clinical reasoning and judgment to guide treatment
	23. Establish, in collaboration with the patient and his/her family, a therapeutic management plan focusing on the patient’s needs: healing, symptom relief, improving function, slowing disease progression, and appropriate end-of-life care
	24. Recognize and rapidly treat patients with acute pathologies ranging from benign to life-threatening situations: trauma, medical, and surgical presentations of illnesses affecting the different body systems, systemic affections (sepsis, shock, altered general state, anorexia, intoxication, CBRN exposure), and psychiatric disorders
	25. Recognize the care specificity of certain populations: pregnant, pediatric, and geriatric patients; immunocompromised patients; and those with cancer, end-of-life, victims of violence and abuse, patients returning from travel, and recent refugees
	26. Be able to explain to patients and their families the risks, benefits, and justification of a proposed diagnostic or therapeutic procedure and obtain their informed consent
	27. Carry out all diagnostic procedures relevant to Emergency Medicine in a skillful and safe manner (abdominal paracentesis, lumbar puncture, joint aspiration, thoracocentesis, fundoscopic exam and fluorescein test of the cornea, 12-lead ECG, venipuncture, and arterial blood gas sampling, point-of-care ultrasound imaging)
	28. Carry out all therapeutic procedures relevant to Emergency Medicine in a skillfull and safe manner (cardiopulmonary resuscitation, airway management, all relevant therapeutic procedures for critically ill patients, peripheral and central vascular access, local anesthesia and procedural sedation, wound repair, extraction of foreign bodies, management of fractures and dislocations, management of normal and complicated deliveries)
	29. Establish a follow-up plan to ensure continuity of outpatient care: follow-up results of investigations, refer patients to appropriate psychosocial services, and organize consultations with specialists and/or the general practitioner
	30. Contribute to the improvement of healthcare quality by recognizing and remediating any incident affecting patient safety
	31. Ensure patient and healthcare provider safety by adopting appropriate protective measures to avoid exposure to infectious agents, chemical, and radiological hazards
Communicator	1. Establish a professional therapeutic relationship with the patient and his/her family, centered on patient needs that are marked by empathy, respect, and compassion
	2. Recognize the values and preferences of patients and their families and pay particular attention to different ethnic, cultural, and social backgrounds which may have an impact on the clinical approach
	3. Adapt the patient’s environment to ensure his/her physical comfort, privacy, safety, and dignity
	4. Be able to use verbal and non-verbal de-escalation techniques when faced with a conflictual situation involving a patient or his/her family; know how to manage emotionally charged conversations
	5. Gather and summarize in an accurate manner the patient’s medical and psycho-social information; or with the patient’s consent, question other sources of information (previous medical records, family members, general practitioner, pre-hospital health providers, or other health professionals)
	6. Explain to patients and their families in a clear, precise, and timely manner the healthcare provided and the diagnostic and therapeutic procedures, and the results, while ensuring that these are properly understood
	7. Be able to disclose to patients and their families, in a tactful manner, all adverse events that have caused prejudice
	8. Explain to a patient and his family, in a clear and precise manner, the risks incurred if he refuses treatment and/or demands to leave the healthcare setting before receiving treatment
	9. Collaborate with patients and their families in a respectful and non-judgmental manner to ensure their full participation in the healthcare plan
	10. Document in an accurate and complete manner all medical records, in paper or electronic format, thus allowing for information to be exchanged
	11. Be able to share medical information in a manner that respects patient confidentiality
Collaborator	1. Establish a positive, collaborative-centered working relationship with other doctors and healthcare professionals
	2. Promote, in a respectful manner, the sharing of responsibilities and decision-making with other doctors or healthcare professionals
	3. Respond positively to any request for help or advice emanating from general practitioners or specialist physicians
	4. Solicit advice from other team members and inform them of the care plan
	5. Be able to communicate in an efficient manner during crisis situations within the emergency department and/or hospital
	6. Use verbal and non-verbal communication strategies to promote mutual understanding, collaboration, and resolve conflicts
	7. Be able to disclose to patients and their families, in a tactful manner, all adverse events that have caused prejudice
Leader	1. Contribute to the improvement of the healthcare system by analyzing and resolving adverse events affecting patient safety and by adapting the clinical practice
	2. Understand and efficiently use software programs applied in the emergency department to access scientific literature and patient data and to elaborate and manage on-call duty schedules
	3. Manage patient flow through the emergency department and allocate healthcare resources appropriately, particularly in the event of a massive and unexpected increase in patient numbers
	4. Demonstrate leadership skills by contributing to the design, implementation, and evaluation of pre-hospital management strategies, collective emergency or disaster management procedures, intra-hospital procedures, and triage protocols
	5. Delegate professional tasks and activities to other members of the team or other health professionals according to their competencies
	6. Demonstrate the ability to act as a team leader by managing time between clinical practice, administrative and financial tasks, human resources management, and scientific and academic contributions, all the while balancing professional and personal life
	7. Demonstrate leadership skills in crisis situations by applying the principles of crisis resource management
	8. Be able to work as a team leader in a harmonious, benevolent, and respectful manner by sharing the vision, plans, and objectives with the other team members
Health Advocate	1. Collaborate with patients and their families in identifying their needs and facilitate access to the necessary resources, whether socioeconomic, legal, or mental services
	2. Facilitate access to psychosocial and/or legal resources when dealing with interpersonal violence, abuse, and neglect of children and of the elderly
Scholar	1. Be able to improve personal scientific knowledge and clinical practice through ongoing training throughout the professional career
	2. Be able to share knowledge and experience by teaching students, colleagues, and other healthcare professionals
	3. Elaborate training programs and teach the techniques and procedures specific to Emergency Medicine
	4. Ensure patient safety when care is provided by students
	5. Provide students with feedback on their learning progression and participate in the assessment of their skill acquisition
	6. Recognize that uncertainties exist in clinical practice and be able to respond appropriately by integrating the principles of evidence-based medicine and using the medical literature in a critical manner
	7. Understand the principles inherent in scientific research and be able to conduct a research program aiming to improve the quality of care in emergency departments
	8. Demonstrate knowledge of the principles of ethics and informed consent in clinical research relevant to Emergency Medicine; be able to assess the risks and benefits for patients
Professional	1. Practice Emergency Medicine in accordance with the rules of medical ethics and deontology by demonstrating honesty, integrity, humility, commitment, compassion, altruism, and respect for patient confidentiality
	2. Demonstrate an ability to behave responsibly by recognizing the ethical problems that arise in the practice of Emergency Medicine and by acting in favor of a patient whose capacity for discernment is limited or impaired
	3. Recognize and react to disrespectful behavior and those contrary to the code of medical ethics and deontology emanating from a colleague or another healthcare professional
	4. Recognize and react to situations where there exists a conflict of interest
	5. Demonstrate adequate professional behavior during oral and written communication
	6. Exhibit commitment to improving the quality of care and patient safety in accordance with the principles of healthcare accreditation
	7. Exhibit commitment to the medical profession by complying with the standards and laws governing the practice of Emergency Medicine
	8. Pay attention to personal well-being, thus ensuring a sustainable clinical practice that lasts throughout the professional career
	9. Be able to grant personal recovery time to attenuate the negative effects of shift work
	10. Be able to deal with violent situations encountered in the emergency department; events that generate negative emotions; and stress linked to decision-making
	11. Be able to identify colleagues in difficulty and offer them support and a response to their needs

During the third Delphi round, the expert panel (n = 11) determined the levels of skill expertise for each of the 78 core competencies, according to the students’ level of training. The median value, an indicator of central tendency, was determined to be the definitive result of the skill expertise level. For each series, the IQR calculated was low, indicating a high concentration of expert votes around the median value. Concerning the competencies in the Medical Expert, Communicator, Collaborator, Health Advocate, Scholar, and Professional roles, the expected skill expertise level for a Beginner was in the majority “Apprentice,” for a Competent, the expected skill expertise level was in the majority “Functional,” and for a Proficient, it was “Experienced.” Only the Leader role showed more variable expected skill expertise levels. For the Beginner, four competencies obtained the expertise level “Observer” and four competencies “Apprentice.” For the Competent, five competencies obtained the expertise level “Apprentice” and three competencies “Functional.” For the Proficient, one competence obtained the expertise level “Apprentice,” four competencies the level “Functional,” and three competencies the level “Experienced.” The final criteria-based assessment grid developed in this study is shown in the Appendices.

## Discussion

The 78 core competencies were validated by the experts at the end of the second Delphi round. All competencies from the central role, Medical Expert, were approved, which is not surprising, given that these competencies are the basic skills required of an EM doctor. It is interesting to note that all competencies from the Communicator, Collaborator, Scholar, and Professional roles were also approved, reflecting the experts’ awareness of the importance of these non-technical skills in the practice of EM nowadays. Concerning the three competencies that did not reach consensus, one competency was from the Leader role and two from the Health Advocate role. These competencies were considered inappropriate for EM doctors, in particular, the Health Advocate competencies: the notion of disease prevention and health promotion, whether at the individual or community level, is considered by the experts as being under the responsibility of front-line General Practitioners rather than that of EM physicians.

The strength of our study is the internal validity of the research. It followed a qualitative research procedure which ensures that the results are scientifically founded. The Delphi method [14-20] was successfully used to reach a consensus of opinions of our group of experts (the ED clinical rotation supervisors from the three French-speaking universities who are geographically dispersed in Belgium). In our study, anonymity was respected, thus avoiding the opinion-leader effect which could have biased the results. Another advantage of the method used is its limited cost. Due to the type of research applied, no insurance was needed. As the study did not interact with patients, no ethical committee board advisory was required. Furthermore, our study attained external validity. The qualitative approach of our work followed a rigorous process similar to that of quantitative research: definition of the research question; choice of the research sample, of the methodology; and, finally, analysis of the data using statistical tools. In each Delphi round, the three universities were represented, which is a major strength of the study. Across one single grid, we were able to develop two distinct processes: the student learning process (how each postgraduate progresses from one stage of learning development to the next) and the evaluation process of EM postgraduates. Our grid not only identifies the skills required but also serves as a guide to the development of these skills throughout the EM postgraduate’s six-year training, assisting students during their advancement from beginner to proficient stages, permitting their to identify the skills that have been achieved and those not yet attained. Local clinical rotation supervisors can identify at the early stages of postgraduates who are struggling and those in need of greater support. Our grid could improve EM postgraduate experiences by providing better feedback and by promoting objective, uniform evaluation, thus leading to greater satisfaction in the curriculum. We have created a practical assessment tool that can be confidently used by any EM clinical rotation supervisor in Belgium, which can be easily adapted to be used by residency programs in other countries.

The Delphi method [14-20], although effective, has its limits. In our study, several experts abandoned the process as the rounds progressed (six for the second round and one for the third). This can be explained by the length of the questionnaires (the initial number of competencies submitted was substantial) and the duration of the procedure (the study occurring over four months), leading to a certain fatigue among the group of participants. In total, 11 out of the initial 30 experts invited completed the study. This small sample may constitute a limiting factor. The lack of debate between experts is another limitation of the method. Opinions were expressed in the free text zones provided, but there was no possibility of developing nor confronting these ideas in an open debate. There exists a potential bias during the recruitment process at the start of the study. We chose the ED clinical rotation supervisors from three French-speaking universities as participants, Dutch-speaking universities, and EM postgraduates were not included in the study.

Further work may be undertaken in the years to come to develop our tool. It would be interesting to have the grid evaluated by the EM postgraduates and the ED clinical rotation supervisors of the Dutch-speaking universities. A pilot project could be launched by introducing the grid to groups of EM postgraduates in one hospital to gather their feedback on how user-friendly the tool is and the eventual modifications needed.

## Conclusions

The objectives of our study have been achieved. We created a practical assessment tool that identifies 78 core competencies required for graduate EM doctors, integrating technical and non-technical skills. By indicating, for each competence, the level of skill expertise expected according to the training level of EM postgraduates, we can identify learner progression during the six-year training period. The evaluation process is now objective, equitable, and uniform and has been acknowledged by ED clinical rotation supervisors from the three French-speaking universities. It can be confidently used by all local clinical rotation supervisors. Our criteria-based assessment grid developed in this study is a referential tool that can be adjusted in the years to come and adapted to EM of the future.

## Figures and Tables

**Figure 2:**
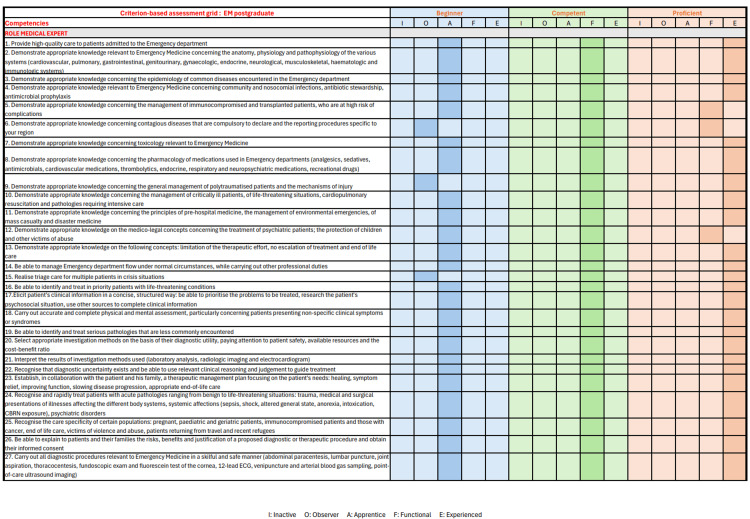
Criteria-based assessment grid: part 1. The darker-colored squares indicate the expected level of skill expertise for each EM postgraduate training level.

**Figure 3:**
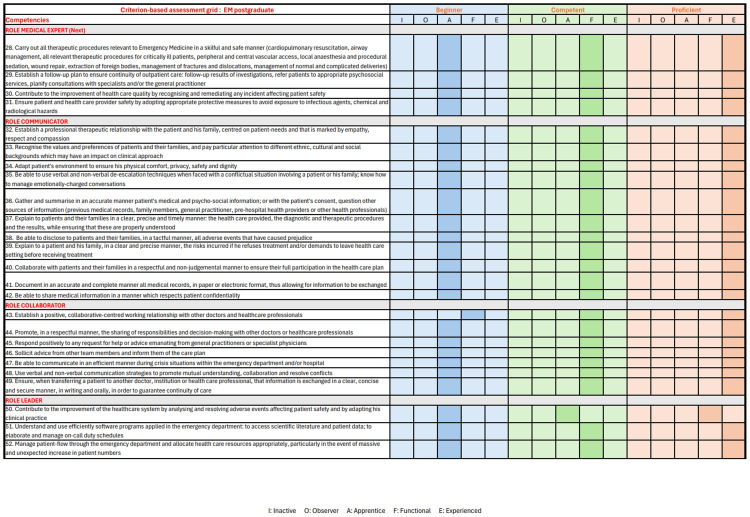
Criteria-based assessment grid: part 2. The darker-colored squares indicate the expected level of skill expertise for each EM postgraduate training level.

**Figure 4:**
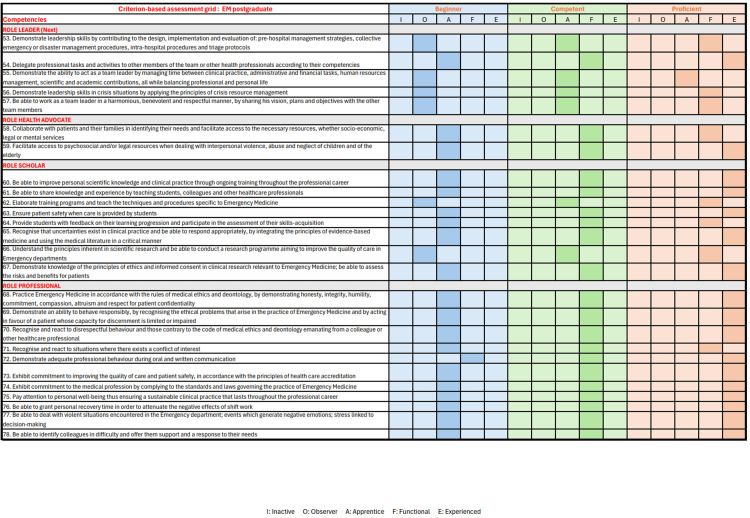
Criteria-based assessment grid: part 3. The darker-colored squares indicate the expected level of skill expertise for each EM postgraduate training level.
